# Decoupling the Arrhenius equation *via* mechanochemistry†
†Electronic supplementary information (ESI) available: Details regarding apparatus design, experimental procedures, and software computations. See DOI: 10.1039/c7sc00538e



**DOI:** 10.1039/c7sc00538e

**Published:** 2017-05-30

**Authors:** Joel M. Andersen, James Mack

**Affiliations:** a Department of Chemistry , University of Cincinnati , 301 Clifton Court , Cincinnati , Ohio 45221-0172 , USA . Email: james.mack@uc.edu

## Abstract

Mechanochemistry continues to reveal new possibilities in chemistry including the opportunity for “greening” reactions. Nevertheless, a clear understanding of the energetic transformations within mechanochemical systems remains elusive. We employed a uniquely modified ball mill and strategically chosen Diels–Alder reactions to evaluate the role of several ball-milling variables. This revealed three different energetic regions that we believe are defining characteristics of most, if not all, mechanochemical reactors. Relative to the locations of a given ball mill's regions, activation energy determines whether a reaction is energetically easy (Region I), challenging (Region II), or unreasonable (Region III) in a given timeframe. It is in Region II, that great sensitivity to mechanochemical conditions such as vial material and oscillation frequency emerge. Our unique modifications granted control of reaction vessel temperature, which in turn allowed control of the locations of Regions I, II, and III for our mill. Taken together, these results suggest envisioning vibratory mills (and likely other mechanochemical methodologies) as molecular-collision facilitating devices that act upon molecules occupying a thermally-derived energy distribution. This unifies ball-milling energetics with solution-reaction energetics *via* a common tie to the Arrhenius equation, but gives mechanochemistry the unique opportunity to influence either half of the equation. In light of this, we discuss a strategy for translating solvent-based reaction conditions to ball milling conditions. Lastly, we posit that the extra control *via* frequency factor grants mechanochemistry the potential for greater selectivity than conventional solution reactions.

## Abstract 2

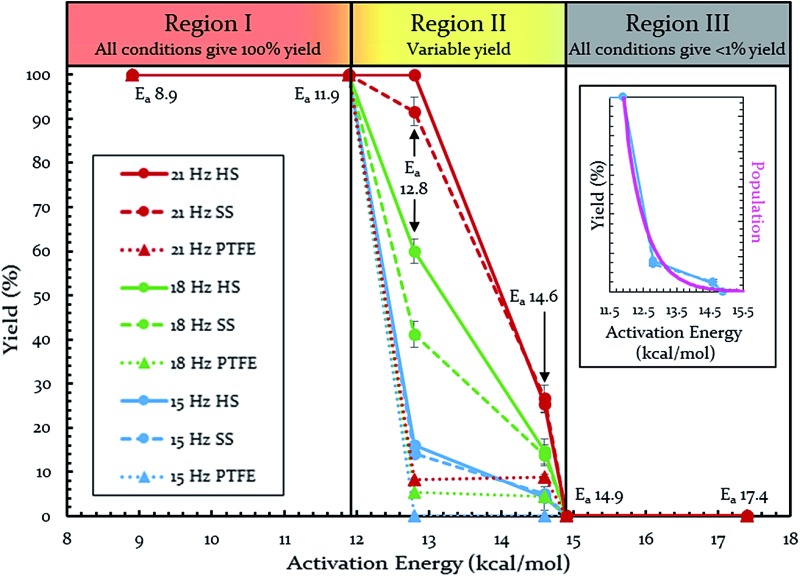
We identified three different energetic regions that we believe are defining characteristics of most, if not all mechanochemical reactions. For a given ball mill's region, activation energy determines whether a reaction is energetically easy (Region I), challenging (Region II), or forbidden (Region III). In Region II, yield depends exponentially on oscillation frequency. Modifications granted control of the locations of Regions I, II, and III.

## Introduction

Mechanochemistry, the field of chemistry relating to mechanically-induced (*i.e.*, grinding or colliding) reactions, has experienced rapid growth within the chemical community.^1–10^ This growth is caused by a mounting interest in exploiting the unique conditions involved. In conventional solvent reactions, solvent choice may heavily influence the possible reaction pathways. Similarly, the absence of solvent in (many) mechanochemical reactions dictates a defined set of pathways too, some of which are unique. Much of the mechanochemistry literature is devoted to exploring these pathways in various fields of chemistry (*e.g.*, inorganic, organic, organometallic, polymeric, *etc.*).^11–22^

Less attention, however, has been devoted to developing an understanding of how the energetics of these systems work and how they can be controlled.^23–27^ This is possibly because there are many methods of inducing mechanochemical reactions, which may all have unique factors involved in their energetics. For example, two seemingly disparate methods of mechanochemical activation are planetary milling and vibrational milling. Planetary milling involves the high-speed rotation of drums containing reactants and reagents, as well as some kind of additional grinding media such as dozens or hundreds of milling balls.^28^ On the other hand, vibrational mills use high-frequency oscillations/shakings of a reaction vial, often with just a single ball, to induce chemistry.^15,27,29–34^ The vial and balls may be made out of inert materials such as stainless steel (SS) or Teflon (PTFE). Interestingly, copper and nickel vials have demonstrated *in situ* catalytic behavior.^19,35^ In addition to the different types of ball mills available (planetary, vibratory, *etc.*) ball mills from different manufacturers can provide different results as well. For example, mixer mills produced by Retsch^36^ (*e.g.*, MM 200 and MM 400) and Fritsch^37^ (*e.g.*, Pulverisette 23) are manufactured with the motor external to the grinding jars. By contrast, the Spex 8000M^38^ produced by SpexCertiprep has the motor encased in the same environment as the grinding jars which adds additional heat to the milling environment. This difference in thermal energy is mainly due to how the ball mill is constructed, which can influence the environment of the mechanochemical reaction.^27,39^

The actual temperature of a milled chemical reaction is still unclear. According to reports in the literature, scientists have reported temperature ranges as low as 40 °C in vibratory mills to as high as 600 °C in planetary mills.^31,40,41^ However, these reports measured the temperature of the ball and/or vial after the reaction concluded, which doesn't provide information on the temperature of the reaction at impact, which has been theorized to be well over 1000 K.^42^ Recently, more sophisticated studies have been performed to get a better understanding of the temperature of milling reactions *in situ*.^27,32,34,39^ Although these are significant improvements over previous results, these methods still cannot provide the chemical energy created at impact. In order to gain more insight into the energetics of milled chemical reactions, especially to gauge how much of the available energy in the milling process is actually transferred to the molecules upon impact, we studied the Diels–Alder reaction under these unique conditions.^43^ Although our previous report provided a little more insight into the energetics of these reactions, the precise role of the ball and its characteristics remained unclear: how is this mechanical force compelling chemistry along? Previously in the literature this question has been addressed with reactions where the rate was significantly dependent on the induction period.^15,27,32,34,39,44^ For example James and coworkers studied the deprotonation of imidazole by zinc oxide to examine the role of oscillation frequency on reaction rate. As they expected, they observed that an increase in oscillation frequency resulted in an increase in the reaction rate for this specific diffusion-limited reaction. The authors envisioned this as resulting from the ball acting as a tool for constantly re-exposing unreacted starting material (*i.e.*, clearing product out of the way such that fresh reagents can interact). However, it is of key importance to determine how this picture holds for reactions that are not diffusion controlled but instead may have a fairly high activation barrier.

We elected to continue exploring the energetics of the 8000M mill using Diels–Alder reactions. The Diels–Alder reaction is uniquely suited to this energy exploration because it involves two reactants creating a single product in a single concerted step, without the need for any other reagents or catalysts. In this way, its use minimizes potential confounding effects. Furthermore, the activation energy of a Diels–Alder reaction can be readily altered in a straightforward manner by changing the substituents on the reactants. This allows us to directly observe how changes in mechanochemical conditions correspond to changes in yield and accessible activation barriers. The intention of this work is to identify and combine the most important variables for ball milling reactions into a concise and well-defined picture, allowing us control of reaction energetics and predictive capabilities. In addition, the results of these experiments may be useful to unify the ball milling community such that we have a method to calibrate ball mills independent of type and manufacturer.

## Results and discussion

**Scheme 1:**
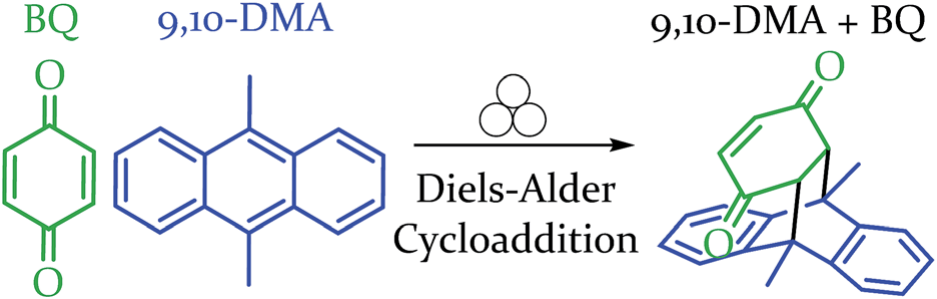
Depiction of the reaction between the diene and dienophile in an example Diels–Alder reaction.

In order to explore both positive and negative effects on energy (and thus yield), we desired a Diels–Alder reaction yielding approximately 50% under our baseline conditions (stainless steel vial oscillating at 18 Hz). The Diels–Alder reaction of benzoquinone (BQ) with 9,10-dimethylanthracene (9,10-DMA), as outlined in Scheme 1, yielded 41% ± 3% of theoretical yield (all errors reported as standard error of the mean, *n* = 3). All reactions were run at a 0.5 mmol scale for three hours (pertinent experimental details for this and other reactions are available in the ESI S2†). With an appropriate reaction in hand, we explored the role of oscillation frequency and vial material/hardness (see ESI S2† for specifics on determining oscillation frequency).

**Fig. 1:**
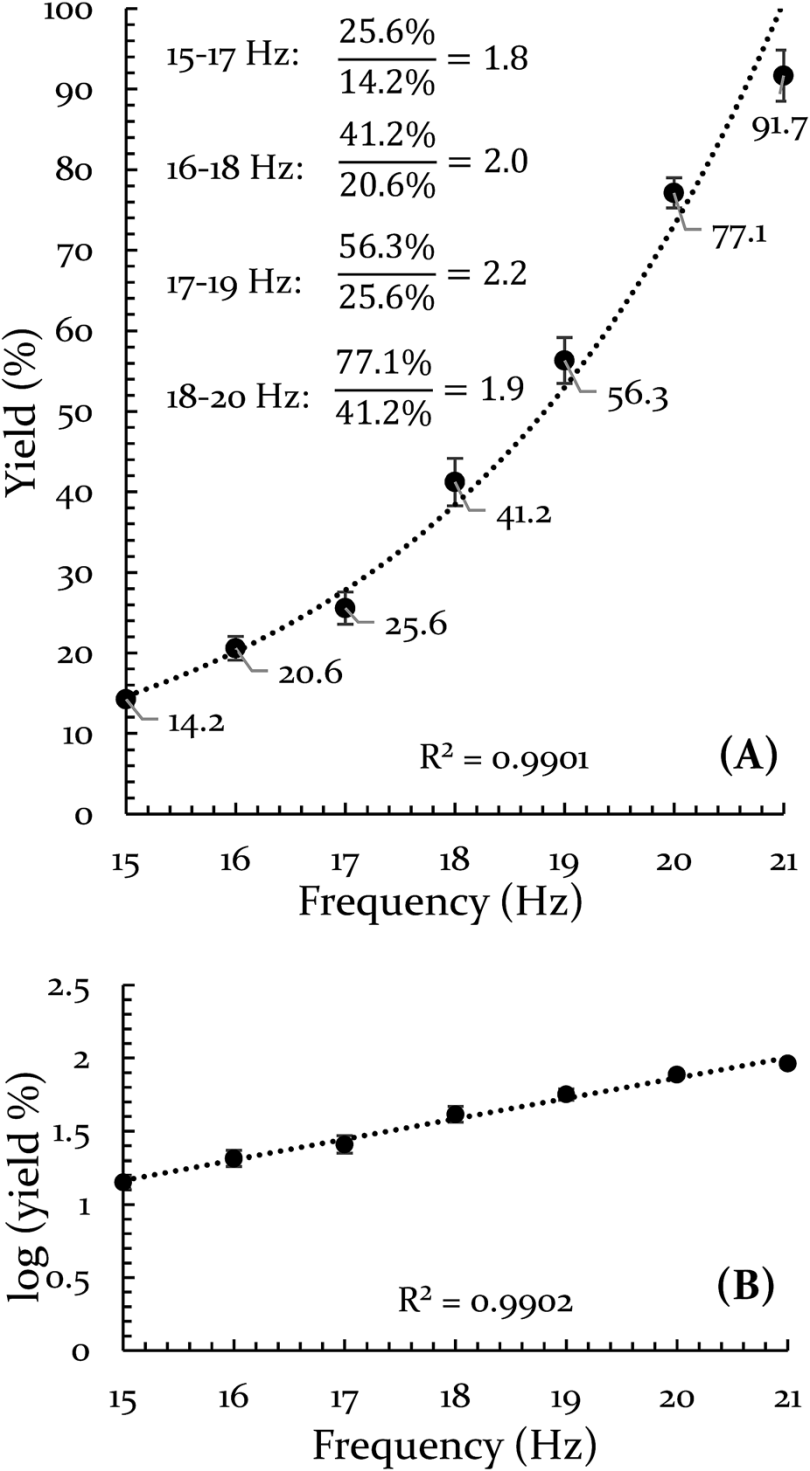
Evidence for an exponential effect of frequency on reaction yield for the Diels–Alder reaction of 9,10-DMA and BQ. (A) Percent yield is plotted *versus* frequency and fit with an exponential curve. (B) The log of the yield is plotted *versus* frequency.

The results of the frequency investigation are presented in Fig. 1. Of critical importance is the observation that the yield doubles every 2 Hz increment over the entire range of tested frequencies. This indicates an exponential dependence on frequency. The doubling effect is reminiscent of the guideline that increasing the temperature of a solution reaction by 10 °C will double the reaction rate. However, the temperature of the vial was monitored throughout the reaction and its average temperature only differed by ∼2 °C when comparing 15 Hz and 21 Hz experiments (details of this measurement are available in the ESI S3†).

**Fig. 2:**
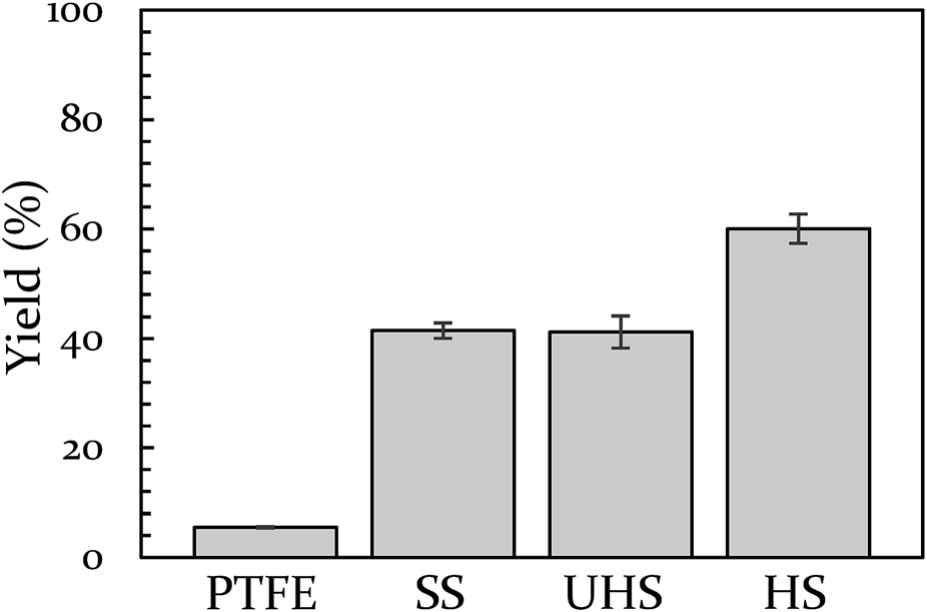
The effect of vial material on yield of 9,10-DMA + BQ at 18 Hz. Teflon (PTFE), unhardened steel (UHS), stainless steel (SS), and hardened steel (HS).

Vial material has also been shown to influence yield. In a prior study, we demonstrated that PTFE vials (soft) produce a lower yield than stainless steel vials (hard).^43^ In the present study we include a control for any potential catalytic behavior of the metal by creating a vial from heat-treatable steel (see ESI S5† for details on this process). The BQ + 9,10-DMA reaction was run again at 18 Hz in Teflon, hardened steel, unhardened steel, and stainless steel vials. This data is presented in Fig. 2. The hardened steel vial produced 60% yield while the unhardened steel and stainless steel vials both produced 40% yield. All three outperform Teflon, which results in only 6% yield. The difference between the hardened vial and unhardened vial isolates the effect of hardness, indicating a significant role. Furthermore, since the unhardened vial produces a yield that is indistinguishable from the stainless steel vial, we opted to perform subsequent comparison experiments in stainless steel instead of the unhardened steel, as they are commercially available.

The underlying cause of the frequency and hardness effects remains unclear. It is possible we are increasing the amount of “chemically usable energy” released during the impact. It is also possible we are merely increasing the rate of molecular collisions between reactants by affecting the mixing. It is also possible we are affecting both. To get some insight on this point, we expanded our data to an entire series of Diels–Alder reactions spanning a range of activation energies.

**Scheme 2:**
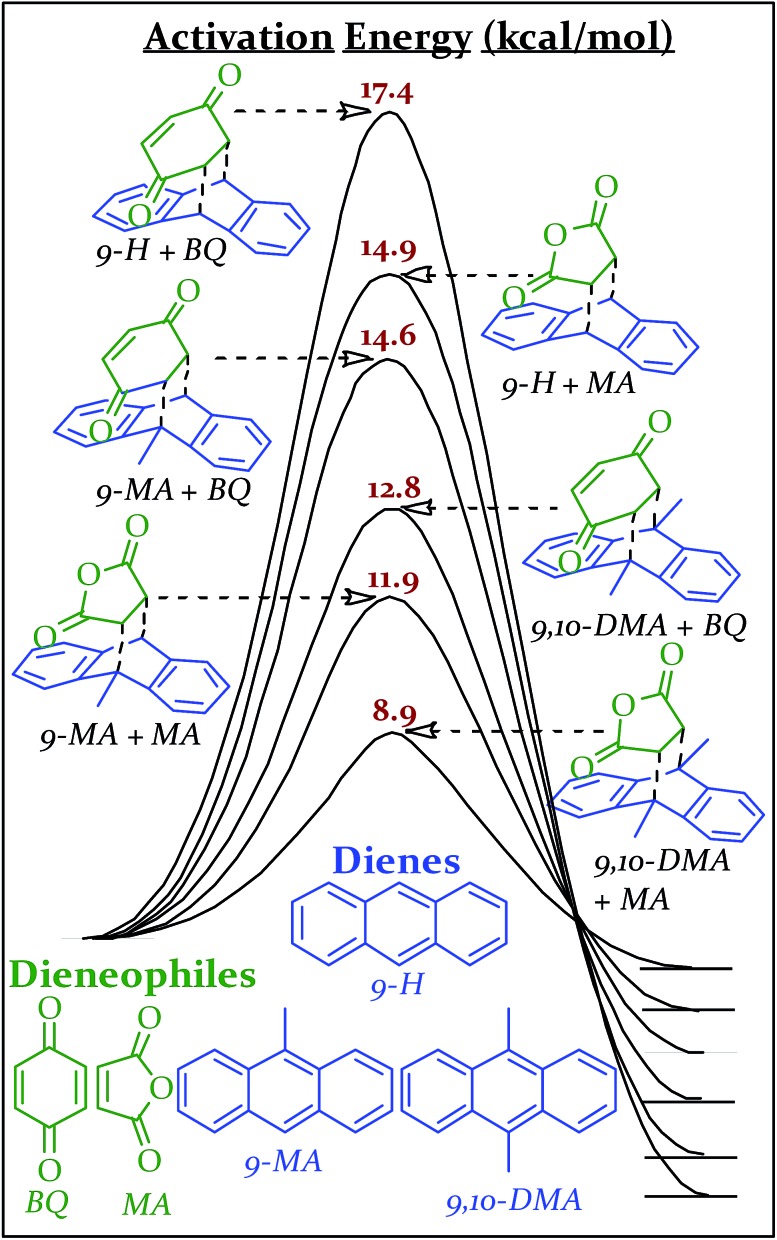
Reaction coordinate diagrams with activation energies of various Diels–Alder reactions as calculated using mPW1PW91/6-31+G(d,p) in Gaussian '09.

The transition state geometries and energies for the Diels Alder series, were calculated using the mPW1PW91/6-31+G(d,p) level of theory and basis set. Linder and Brinck studied the effect of theory and basis set on Diels–Alder transition state calculations and found good agreement (generally ±1.5 kcal mol^–1^) with benchmark CCSD(T)/6-31+G(d)//CCSD/6-31+G(d) calculations obtained with the Gaussian 09 program suite.^45–48^ Two of our calculated transition state energies (9,10-DMA + MA and 9-H + MA) have been previously determined experimentally in xylenes and match within 1.0 kcal mol^–1^.^49^ The combinations of these reactants (all solids at the vial's temperature during milling) produce six different Diels–Alder adducts, all with different activation energies (Scheme 2). Computational details are available in the ESI S5.†

**Fig. 3:**
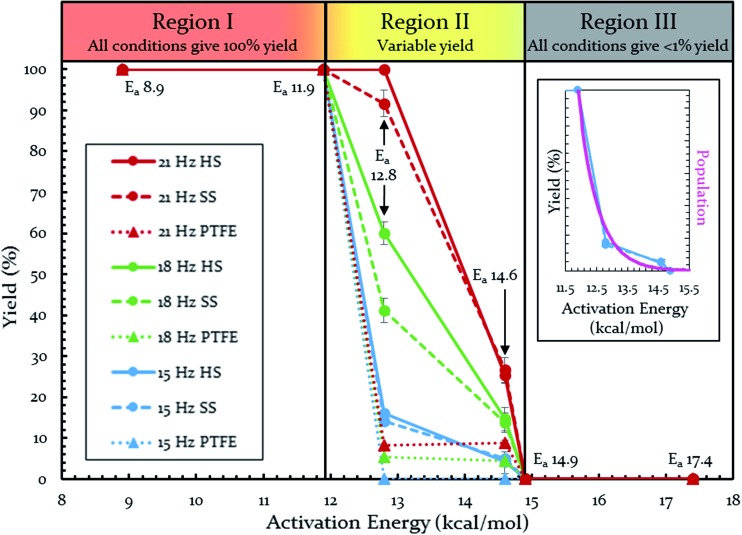
Yields of Diels–Alder reactions under various ball-milling conditions. Note: all nine conditions produce overlapping yields for *E*_a_ 8.9, 11.9, 14.9, and 17.4 kcal mol^–1^. Individual plots are available in the ESI (S14†). Teflon (PTFE), stainless steel (SS), and hardened steel (HS). Inset: population of energies determined by Boltzmann distribution overlaid on top of yield data.

Each of these six reactions were subjected to nine different ball-milling conditions (permutations of various vial materials and oscillation frequencies). Fig. 3 contains these results (note that the 18 Hz data for *E*_a_ = 12.8 kcal mol^–1^ was present in Fig. 1 and 2). For ease of reference in discussing the results, we gave the label “Region I” to the energy range encompassing reactions that, regardless of the frequency (*i.e.* 15–21 Hz) we chose or vial material used (*i.e.* Teflon, stainless steel, hardened steel). On the opposite end of the figure, we gave the label “Region III” to the energy range containing reactions that produced no observable yield after three hours of milling, regardless of frequency chosen or vial material used (we extended reactions times to 16 hours and still didn't observe any product). We labeled the intermediate range “Region II.” Note that the positions of Regions I, II, and III are not universally fixed. Indeed, they are a function of several variables, such as how long the reactions were run, but in practice they are useful as they highlight the area of greatest sensitivity to mechanochemical conditions (Region II). The reactions associated with Region III are theoretically attainable given significantly longer milling times, but from a practical perspective, we are focusing reaction times that are applicable to conventional ball mills. Furthermore, given the differences in ball mills, type and manufacturer, it is important to be able to develop a nomenclature that universally describes the limitations of unmodified ball mills.

Regions I, II, and III display noteworthy characteristics that can be explained by an argument rooted in the Arrhenius equation (eqn (1)). First, we must appreciate that even if the ball wasn't present, the molecules would still presumably be in equilibrium with the temperature of the vial itself. This temperature is 36 °C for all six reactions conducted at an oscillation frequency of 15 Hz. As mentioned previously, when changing the frequency from 15 Hz to 21 Hz, the temperature of the vial increases by just 2 °C. With this in mind, one could argue that in “Region I” (*E*_a_ ≤ 11.9 kcal mol^–1^), a large enough fraction of molecules possess enough energy that we observe quantitative conversion even when the collisions between molecules are at the minimum for our conditions (PTFE and 15 Hz, where you may imagine the mixing caused by the ball is very inefficient). Thus, in the case of “Region I” we are led to assume that the energy profile term (e^–*E*_a_/*RT*^) of eqn (1) is sufficiently large such that relatively few collisions are needed to reach quantitative yield. In “Region II” (11.9 kcal mol^–1^ < *E*_a_ < 14.9 kcal mol^–1^), we could attribute increases in yield to increases in frequency factor (“*A*”) due to improved mixing as we use harder materials or higher frequencies. Therefore, although the energy profile term is less favorable due to a higher activation energy, it can be offset by an increased collision rate/frequency factor (*i.e.*, speeding up the mill). “Region III” encompasses reactions that are not accessible given practical frequency limitations on our ball mill and our given time frame. Thus, we propose calling Region I a “thermally-driven region,” Region II a “collisionally-driven region,” and Region III a “energetically limited region.”1
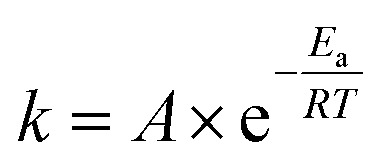



Since the main energy source of these reactions would come from the overall temperature of the vial, this theory predicts that an overlay of a theoretically calculated Boltzmann (or Boltzmann-like) distribution should correlate reasonably well with the observed results. This suggests that oscillation frequency and hardness would merely act upon the thermal distribution. This overlay (assuming *R* = 0.001986 kcal K^–1^ mol^–1^) is presented in Fig. 3's inset, demonstrating outstanding agreement. Furthermore, comparing this to “Region II” in Fig. 3 well delineates the additional benefit of the increased collision rate acting on the thermally available energy. In effect, this means controlling *A* in the Arrhenius equation. It is important to note that an equivalent control over collision rate cannot be had in well-stirred solutions (*e.g.*, solutions with no gradient in concentration or temperature).

**Fig. 4:**
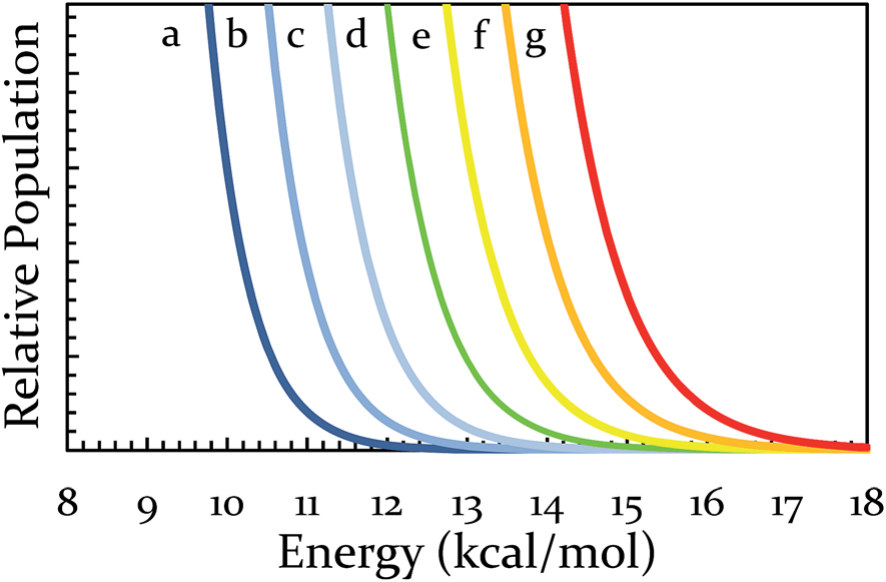
Calculated Boltzmann distributions for various temperatures. Line d represents the vial temperature in our unmodified mill (∼36 °C). Lines a–c and e–g represent the effect of ±20 °C increments away from that temperature.

At this point, one can see that a ball-milling approach to solvent-free reactions affords chemists separate access points to reaction rates: thermal and collisional. Theoretically speaking, the upper end of the collisional frequency effect may be limited only by the lifetime of molecular vibrations. However, the current engineering and design of ball mills forces a practical upper limit to the maximum collisional frequency (*i.e.*, when we attempted 22 Hz for extended times the milling apparatus broke, other mills may differ in frequency limitations). Because there are currently practical limitations on *A*, we investigated the effect of changes in the system's temperature on the rate of reaction. We hypothesized that changing the vial temperature will let us choose between the Boltzmann distributions in Fig. 4. Shrewd selection of Boltzmann distribution should effectively control the locations of Regions I, II, and III, assuming the milling time is unchanged.

To this end, we made several modifications to the ball mill. For testing the effect of a temperature decrease, the ball mill was interfaced with a cooling unit (see ESI S3† for details). The yield of BQ + 9,10-DMA served as a comparison point. Under normal operating conditions using a hardened vial oscillating at 21 Hz the peak operating temperature was 38 °C. When cooled during operation, the system maintained a peak operating temperature of about 22 °C. This modest drop of 16 °C caused the yield to plummet from 92% to 12%. The significance of this result cannot be overstated. Because oscillation frequency was unchanged, it is reasonable to attribute the drop in yield to indicate a significant dependence on temperature as opposed to any change in the pre-exponential factor. This greatly bolsters the Arrhenius-based argument. Simultaneously, it suggests that for an activation energy of 12.8 kcal mol^–1^, the impact of the ball with the wall does not provide a significant amount of chemically usable energy. The current method for cooling is undesirable for practical purposes, but has been useful as a proof of concept. Development of an improved cooling method with access to all Boltzmann distributions in Fig. 4 is currently in progress.

**Fig. 5:**
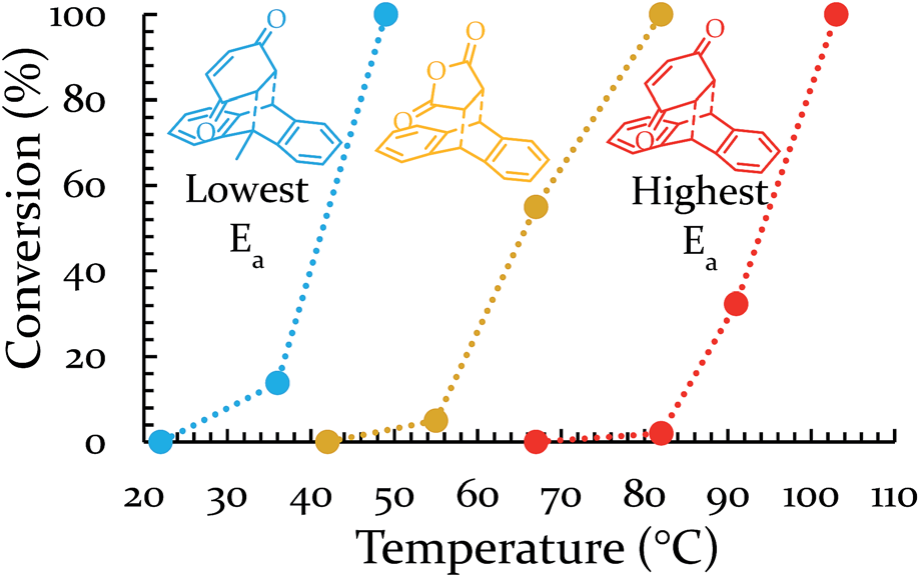
Dependence of percent conversion on vial temperature.

Having decreased the temperature of the system, increasing the temperature is important not only as another test of the theory, but also because it would shift all regions towards higher energies. Thus, we would obtain access to reactions that were previously inaccessible to us on a reasonable timescale. To increase the temperature, a heating band (BB010004, http://www.instrumentation-central.com/) was wrapped around an aluminum rod holding the vial. Coupling the band with a Variac allowed temperature control of the vial with a fair amount of precision (±2 °C) at any targeted temperature. See ESI S3† for details regarding the reproducibility and reliability of this setup. The two reactions of the conditionally-forbidden region (III) (and a third reaction for which the mill could not reach quantitative yield in 3 h) were each tested at a variety of temperatures. The results of these experiments (18 Hz, SS) are displayed in Fig. 5. All three reactions now readily reach quantitative conversion within three hours. These results make clear that we have successfully identified a way to shift the regions of Fig. 3 to higher activation energies. For the sake of comparing with solution, the reaction of 9-H + BQ (*E*_a_ = 17.4 kcal mol^–1^) is done at ∼140 °C (refluxing xylenes), and we observed quantitative conversion at just 100 °C.^50^ We expect that in the solvent-free ball milling conditions here, reactants are free of fruitless collisions with solvent molecules, allowing a significant boost in reaction rate. Lastly, it is important to note that the milling and heating must occur simultaneously, as we observed in a separate experiment that if the 9-H + BQ reaction is milled for three hours with no extra heating followed by subsequent heating in an oven for three hours at a comparable temperature, the yield drops from 100% to 19%.

Given the resounding success of the Arrhenius equation when applied to a vibratory ball mill, we propose a straightforward path for energetically converting conventional, solution-based syntheses to ball mill ones. If selectivity is not a concern, the vial temperature should be high enough to expand “Region I” to encompass the reaction. If selectivity is needed to avoid side reactions, “Region II” should be targeted. Once a reaction is in the collisionally-driven region (Region II), fine adjustments can be made such as a change in oscillation frequency or vial and ball material, which will have a significant effect on reaction rate and selectivity.

## Conclusions

Mechanochemistry has continued to grow in popularity despite a poor understanding of the inner workings of its energetics. Our experiments suggest that we can conceptualize vibrational ball mills (and likely many other forms of mechanochemistry) as collision-facilitating devices that act upon molecules existing in a thermally-derived energy distribution. In this way, conducting chemical reactions in a variable-frequency, variable-temperature ball mill allows chemists to decouple both halves of the Arrhenius equation: the frequency factor (“*A*”) and the energy profile term (e^–*E*_a_/*RT*^). Coarse adjustment of temperature and optional fine adjustment of oscillation frequency dictate the energetics and thus serve as a way to translate established conventional syntheses to ball mill conditions. This proposal is straightforward, yet powerful in its predictive capabilities. It has succeeded in getting access to reactions that were previously inaccessible in the mill due to prohibitively high activation energies.

Finally, we would like to argue heuristically that the new access point to the frequency factor grants mechanochemistry a unique prospect with respect to selectivity. Conventionally, a chemist decreases temperature to increase selectivity, a result of narrowing the Boltzmann distribution. However, it may be the case that in the process of obtaining the desired selectivity, that either (A) the rate of the target reaction is slowed so much as to render the reaction impractical or (B) solubility problems arise. At this point, the chemist would require a catalyst, which may be toxic, expensive, and/or inconvenient. In the ball mill, however, our theory predicts that the selectivity should be unchanged by oscillation frequency. In this way, we can recover the original reaction rate by increasing the oscillation frequency without sacrificing selectivity. Maximum selectivity would be observed by operating the highest practical oscillation frequency upon the lowest practical temperature to produce an acceptable reaction rate. We are currently investigating this possibility.

## Conflict of interest

The authors declare no competing financial interest.
