# Supplementary material for: Decoupling the Arrhenius equation via mechanochemistry
Source: Chem Sci. 2017 May 30;8(8):5447–53. doi: 10.1039/c7sc00538e (PMC5609516; doi:10.1039/c7sc00538e)

Electronic Supplementary Material (ESI) for Chemical Science.
This journal is © The Royal Society of Chemistry 2017

Decoupling the Arrhenius Equation via Mechanochemistry

Joel Andersen, James Mack

Department of Chemistry, University of Cincinnati, 301 Clifton Court, Cincinnati, Ohio 45221-0172, United states

Supporting Information

Table of Contents

1.	**Reaction Procedure**	**S2**
2.	**Determination of Oscillation Frequency**	**S2**
3.	**Vial Temperature Measurement and Control**	**S3**
4.	**Hardened Vial Production**	**S5**
5.	**Computation Parameters and x, y, z Coordinates**	**S5**
6.	**Individually Plotted Data from Figure 3**	**S14**

Reaction Procedure

Typical Reaction Procedure (9-H + BQ example). To a stainless steel vial, anthracene (0.0890 g, 0.500 mmol) and maleic anhydride (0.0490 g, 0.500 mmol) were added. A 3/16 inch stainless steel ball was then added. A Teflon o-ring was included to ensure proper seal between vial and cap. The vial was clamped in a Spex 8000M mixer/mill and reacted for three hours. All chemicals were purchased from Acros Organics except for 9,10-dimethylanthracenem, which was purchased from TCI Chemicals. All reactions were carried out with the same batch of chemicals.

Analysis. Reactions were first analyzed by removing a small sample directly from the vial for H NMR analysis. While convenient, it is also important because some of these reactions will proceed readily at the high concentrations and (potentially) elevated temperatures involved in rotatory evaporation. Considering the importance of obtaining accurate yields, such conditions must be avoided. Reactions producing 0% or 100% conversion by H-NMR were not analyzed further. Remaining reactions were analyzed by high-performance liquid chromatography (HPLC). First, vial contents were extracted with dichloromethane (DCM) and diluted further with DCM to 100 mL using a volumetric flask. After thorough mixing, 0.25 mL was removed using a disposable 1 mL syringe. This 0.25 mL sample was added to 5.00 mL of acetonitrile (ACN). This was thoroughly mixed and 1 mL was removed for HPLC analysis on an Agilent 1100 Series HPLC. All analyses were performed using a Phenomenex Synergy Hydro RP 150x4.6mm, 4μ column. The reaction between BQ and 9,10-DMA was analyzed accordingly: flow rate 1.1 mL/min, injection volume 10 μL, column temperature 21.5 °C, wavelength monitored 278 nm, solvent system 75% H_2O, 25% ACN. The reaction of BQ with either 9-MA or 9-H was the same as above with the following modifications: wavelength monitored 210 nm, solvent system 32% H_2O, 68% ACN. Calibration curves allowed back calculation of yield.

Determination of Oscillation Frequency

Two key variables go into the oscillation frequency: the motor's operating frequency and pulley ratio connecting the motor to the shaft of the mill. The mill that we used (Spex 8000M Mixer/mill, http://www.SPEX SamplePrep.com) is by default a single oscillation-frequency mill. However, replacing the stock motor with a Marathon Y502 motor) and interfacing that motor with a Frenic Mini Inverter (Fuji Electric FRNF50C1S-6U) allows easy control of the motor's operating frequency. The mill's front metal lid was replaced with a homemade clear

plexiglass cover to allow direct observation of the milling process. From this observation, an accurate measure of the oscillation frequency was obtained using high-speed film. There was a strong linear correlation between the setting on the inverter's potentiometer and the operating frequency. For example, settings of 50, 60, and 70 Hz on the potentiometer produced oscillation frequencies of 14.5, 17.4, and 20.2 Hz. The ratio between the potentiometer setting and the oscillation frequency for these three settings are 3.45, 3.45, and 3.46, respectively. As a result, we could easily target a frequency (e.g., 16 Hz) by setting the potentiometer to the product of the frequency and 3.45.

Vial Temperature Measurement and Control

The temperature of the vial was determined by use of a temperature logging "iButton" (DS1922E-F5#, Maxim Integrated Circuits, http://www.ibutton.com) clamped between the top of the vial and the clamp assembly. This temperature rises for approximately the first hour and then maintains for the remainder of the experiment. An example heating pattern with no external heating/cooling is provided in Supporting Figure 1. The reaction was cooled by forcing cold air over the entire mill. A Frigidaire window cooling unit (Model #LRA087AT7) was coupled to the mill via flexible duct as shown in Supporting Figure 2. The temperature curve for this is presented in Supporting Figure 1. Minor fluctuations are inevitable due to the cooling unit, but for its intended purpose it worked quite well. To heat the vial, a thermal band (BB010004, http://www.instrumentation-central.com) was wrapped around an aluminum rod holding the vial. The aluminum rod was designed to encase the entire vial (and two iButtons) and also to have a snug fit with the thermal band. It was important to have it symmetrical to avoid uneven heating of the vial. The heating band, aluminum holder, and vial are all pictured in Supporting Figure 3. A typical heating trend is presented in Supporting Figure 4. The average of the top and bottom iButtons (once steady) was reported as the vial temperature. Temperatures were reproducible within approximately ±1 °C.

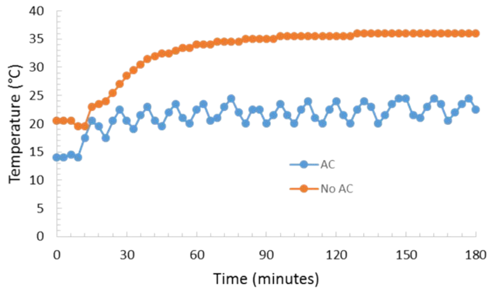

Supporting Figure 1. "AC" stands for air conditioning (of the mill).

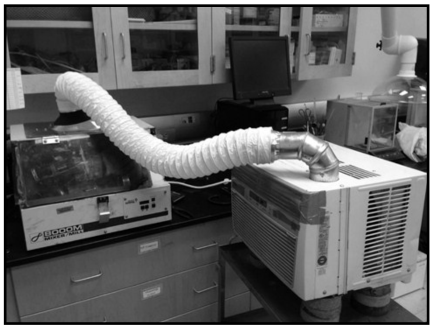

Supporting Figure 2.

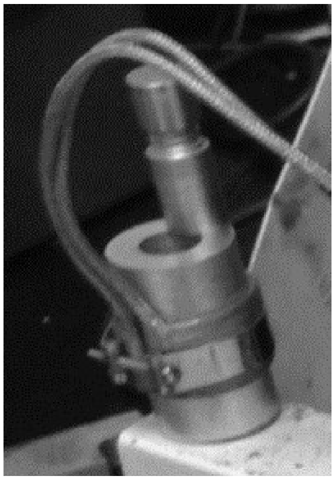

Supporting Figure 3.

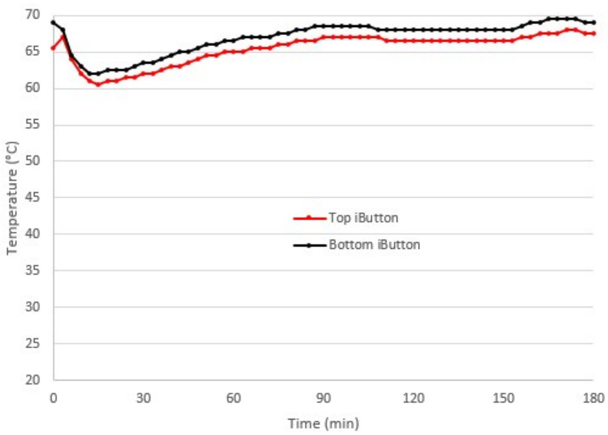

Supporting Figure 4.

Hardened Vial Production

Prior to heat-treating, the raw stock material (S7 heat-treatable steel, rod, https://www.mcmcaster.com) was shaped using a metal lathe. Two vials were shaped, but one was left un-treated (to serve as control). The treated vial was heated in a programmable furnace (HT-22-D, Thermcraft) according to the specifications provided by the manufacturer to achieve the hardest vial possible after tempering at 400 °F. Three hardness measurements were averaged and the Rockwell C Hardness was determined to be C51 (control vial was Rockwell B Hardness B79). The reaction of BQ with 9,10-DMA were run in the control vial in triplicate, yielding 41.5% (±1.4%, standard error of the mean). When performed in a stainless steel vial we observed a yield of 41.2% (±2.9%). This overlap suggests that stainless steel can be compared directly with the hardened vial despite potentially having minor differences in atomic make up. A schematic of the vial design is provided below. Teflon vials are made to the same dimensions, but no threads are included as they are too fragile.

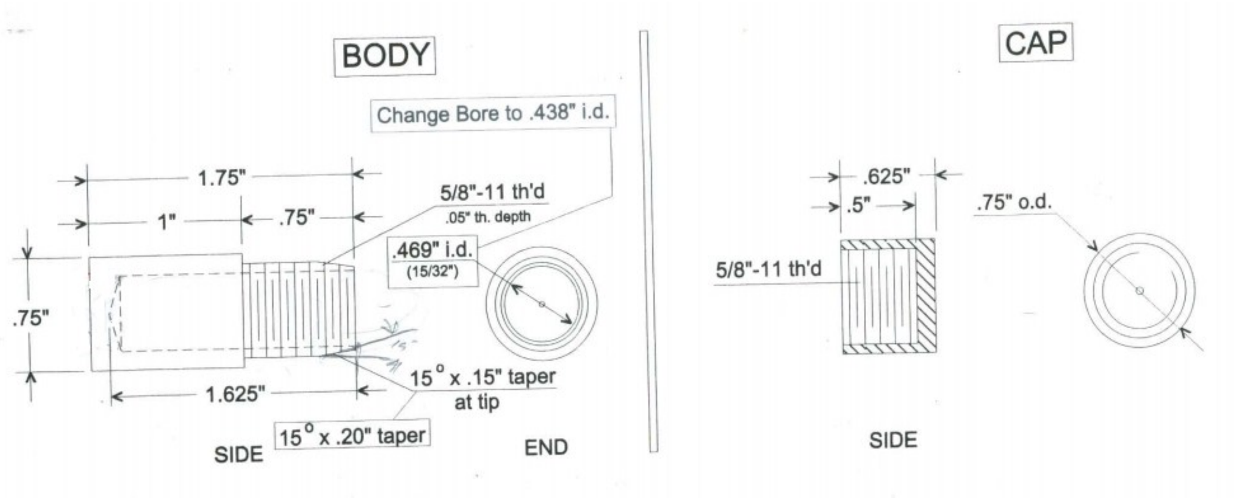

Computation Parameters and x, y, z Coordinates

Anthracene (9-H)

opt mpw1pw91/6-31+g(d,p) geom=connectivity

Center Number	Atomic Number	Atomic Type	Coordinates (Angstroms) X	Y	Z
1	1	0	-4.596457	1.244674	0.000002
2	6	0	-3.651564	0.711653	0.000001
3	1	0	-2.470498	2.489878	0.000000
4	6	0	-2.472592	1.404055	0.000000

5	6	0	-2.472592	-1.404055	0.000000
6	6	0	-1.220360	0.719735	-0.000001
7	6	0	-3.651564	-0.711653	0.000001
8	6	0	-1.220360	-0.719735	-0.000001
9	6	0	0.000000	1.400763	0.000000
10	1	0	-4.596457	-1.244674	0.000002
11	1	0	0.000000	-2.487454	0.000000
12	1	0	-2.470498	-2.489878	0.000000
13	6	0	1.220360	0.719735	-0.000001
14	6	0	2.472592	1.404055	0.000000
15	6	0	1.220360	-0.719735	-0.000001
16	1	0	2.470498	-2.489878	0.000000
17	6	0	0.000000	-1.400763	0.000000
18	6	0	3.651564	0.711653	0.000001
19	1	0	2.470498	2.489878	0.000000
20	1	0	4.596457	1.244674	0.000002
21	6	0	3.651564	-0.711653	0.000001
22	1	0	4.596457	-1.244674	0.000002
23	6	0	2.472592	-1.404055	0.000000
24	1	0	0.000000	2.487454	0.000000

9-methylanthracene (9-MA)

opt mpw1pw91/6-31+g(d,p) geom=connectivity

Center Number	Atomic Number	Atomic Type	Coordinates (Angstroms) X	Y	Z
1	1	0	-4.573397	-1.551486	0.000002
2	6	0	-3.639355	-0.999764	0.000001
3	1	0	-2.415449	-2.748608	0.000001
4	6	0	-2.444579	-1.663092	0.000000
5	6	0	-2.490533	1.134337	-0.000002
6	6	0	-1.207024	-0.953681	0.000000
7	6	0	-3.658554	0.420253	0.000000
8	6	0	-1.217418	0.484650	-0.000001
9	6	0	0.013223	-1.628672	0.000000
10	1	0	-4.609707	0.942379	0.000000
11	1	0	-2.536432	2.216534	-0.000003
12	6	0	1.222627	-0.936766	0.000000
13	6	0	2.464039	-1.641777	0.000001
14	6	0	1.223875	0.506994	-0.000002
15	1	0	2.555995	2.234857	0.000000
16	6	0	-0.002731	1.206711	-0.000002
17	6	0	3.656533	-0.977321	0.000000
18	1	0	2.436331	-2.727316	0.000002
19	1	0	4.592304	-1.526003	0.000000
20	6	0	3.669324	0.443221	0.000000
21	1	0	4.618041	0.969690	-0.000001
22	6	0	2.500767	1.154042	0.000000
23	1	0	0.021797	-2.715192	0.000002
24	6	0	-0.061288	2.711169	0.000002
25	1	0	-0.592452	3.084206	-0.881495
26	1	0	0.921946	3.174913	-0.000016

27	1	0	-0.592418	3.084201	0.881523

9,10-dimethylanthracene (9,10-DMA)

opt mpw1pw91/6-31+g(d,p) geom=connectivity

Center Number	Atomic Number	Atomic Type	Coordinates (Angstroms) X	Y	Z
1	1	0	-1.251762	4.595731	-0.161954
2	6	0	-1.243005	3.512429	-0.103103
3	1	0	0.866192	3.409154	-0.061499
4	6	0	-0.054461	2.840314	-0.039045
5	6	0	-2.455336	1.428861	-0.038939
6	6	0	0.006799	1.412589	0.026172
7	6	0	-2.464610	2.794254	-0.103048
8	6	0	-1.237609	0.680994	0.026194
9	6	0	1.237609	0.727602	0.073842
10	1	0	-3.406930	3.328720	-0.161873
11	1	0	-3.400110	0.901051	-0.061318
12	6	0	1.237609	-0.680994	0.026194
13	6	0	2.455336	-1.428861	-0.038939
14	6	0	-0.006799	-1.412589	0.026172
15	1	0	-0.866192	-3.409154	-0.061499
16	6	0	-1.237609	-0.727602	0.073842
17	6	0	2.464610	-2.794254	-0.103048
18	1	0	3.400110	-0.901051	-0.061318
19	1	0	3.406930	-3.328720	-0.161873
20	6	0	1.243005	-3.512429	-0.103103
21	1	0	1.251762	-4.595731	-0.161954
22	6	0	0.054461	-2.840314	-0.039045
23	6	0	2.534638	1.490148	0.146726
24	1	0	2.929659	1.722310	-0.849288
25	1	0	3.301244	0.929494	0.681620
26	1	0	2.417530	2.432686	0.681531
27	6	0	-2.534638	-1.490148	0.146726
28	1	0	-2.929659	-1.722310	-0.849288
29	1	0	-3.301244	-0.929494	0.681620
30	1	0	-2.417530	-2.432686	0.681531

Maleic Anhydride (MA)

opt 6-31+g(d,p) geom=connectivity mpw1pw91

Center Number	Atomic Number	Atomic Type	Coordinates (Angstroms) X	Y	Z
1	6	0	0.000000	0.666557	-1.255434
2	1	0	0.000000	1.361049	-2.083332

3	6	0	0.000000	-0.666557	-1.255434
4	1	0	0.000000	-1.361049	-2.083332
5	6	0	0.000000	-1.123218	0.158723
6	6	0	0.000000	1.123218	0.158723
7	8	0	0.000000	0.000000	0.965451
8	8	0	0.000000	-2.233326	0.600224
9	8	0	0.000000	2.233326	0.600224

Benzoquinone (BQ)

opt mpw1pw91/6-31+g(d,p) geom=connectivity

Center Number	Atomic Number	Atomic Type	Coordinates (Angstroms) X	Y	Z
1	6	0	0.000000	0.000000	1.438510
2	6	0	0.000000	1.266839	0.670336
3	1	0	0.000000	2.179571	1.256987
4	6	0	0.000000	1.266839	-0.670336
5	1	0	0.000000	2.179571	-1.256987
6	6	0	0.000000	0.000000	-1.438510
7	6	0	0.000000	-1.266839	-0.670336
8	1	0	0.000000	-2.179571	-1.256987
9	6	0	0.000000	-1.266839	0.670336
10	1	0	0.000000	-2.179571	1.256987
11	8	0	0.000000	0.000000	2.659716
12	8	0	0.000000	0.000000	-2.659716

9-H + BQ

opt=(calcfc,ts) freq mpw1pw91/6-31+g(d,p) geom=connectivity

Center Number	Atomic Number	Atomic Type	Coordinates (Angstroms) X	Y	Z
1	6	0	2.536055	2.427571	-0.712344
2	1	0	3.338862	2.919594	-1.251033
3	6	0	2.536089	2.432068	0.696798
4	1	0	3.338919	2.927525	1.232297
5	6	0	1.526938	1.805665	1.397569
6	1	0	1.532638	1.793271	2.482873
7	6	0	0.459146	1.194783	0.705050
8	6	0	0.459088	1.190292	-0.712585
9	6	0	1.526853	1.796738	-1.409048
10	1	0	1.532464	1.777464	-2.494252
11	6	0	-0.600738	0.474601	-1.353905

12	1	0	-0.563775	0.366512	-2.434957
13	6	0	-1.878406	0.406932	-0.709470
14	6	0	-1.878320	0.411359	0.707097
15	6	0	-0.600545	0.482950	1.350940
16	1	0	-0.563543	0.381832	2.432667
17	6	0	-3.092315	0.237598	1.401860
18	1	0	-3.088198	0.226890	2.487426
19	6	0	-4.275506	0.102675	0.704337
20	1	0	-5.211489	-0.005105	1.241818
21	6	0	-4.275594	0.098284	-0.704493
22	1	0	-5.211644	-0.012850	-1.241175
23	6	0	-3.092494	0.228858	-1.402992
24	1	0	-3.088505	0.211373	-2.488469
25	6	0	0.000674	-1.574775	-0.698519
26	1	0	-0.840668	-2.002344	-1.231848
27	6	0	0.001077	-1.569936	0.709071
28	1	0	-0.839740	-1.994345	1.245753
29	6	0	1.274794	-1.612107	-1.443220
30	6	0	2.517646	-1.394911	-0.666887
31	1	0	3.426605	-1.279311	-1.248375
32	6	0	2.518087	-1.390377	0.674600
33	1	0	3.427419	-1.270842	1.254705
34	6	0	1.275740	-1.602259	1.453183
35	8	0	1.322976	-1.816282	-2.650409
36	8	0	1.324654	-1.798184	2.661699

9-H + MA (TS)

opt=(calcfc,ts) freq mpw1pw91/6-31+g(d,p) geom=connectivity

Center Number	Atomic Number	Atomic Type	Coordinates (Angstroms) X	Y	Z
1	6	0	-2.701500	2.229069	-0.704880
2	1	0	-3.535772	2.668247	-1.240799
3	6	0	-2.701500	2.229157	0.704635
4	1	0	-3.535774	2.668400	1.240499
5	6	0	-1.659688	1.660671	1.404687
6	1	0	-1.670006	1.635515	2.489214
7	6	0	-0.561562	1.110723	0.709474
8	6	0	-0.561566	1.110626	-0.709578
9	6	0	0.525902	0.444133	1.354130
10	1	0	0.492701	0.348457	2.436624
11	6	0	1.803841	0.405592	0.708563
12	6	0	1.803836	0.405499	-0.708602
13	6	0	3.022449	0.259309	1.402391
14	1	0	3.019750	0.248744	2.488031
15	6	0	4.207944	0.153135	0.704552
16	1	0	5.146450	0.066639	1.241405
17	6	0	4.207939	0.153043	-0.704573
18	6	0	-1.659690	1.660489	-1.404860
19	1	0	-1.670010	1.635187	-2.489383

20	1	0	5.146442	0.066478	-1.241420
21	6	0	0.525885	0.443940	-1.354159
22	1	0	0.492670	0.348131	-2.436640
23	6	0	3.022439	0.259124	-1.402419
24	1	0	3.019733	0.248419	-2.488057
25	6	0	0.012841	-1.637243	-0.698787
26	1	0	0.781419	-2.046659	-1.337650
27	6	0	0.012823	-1.637167	0.698983
28	1	0	0.781388	-2.046494	1.337919
29	6	0	-1.397769	-1.684203	1.130666
30	6	0	-1.397746	-1.684326	-1.130496
31	8	0	-2.196899	-1.602327	0.000071
32	8	0	-1.870217	-1.769378	2.229880
33	8	0	-1.870166	-1.769606	-2.229714

9-MA + BQ (TS) (Note: Cartesian keyword required for successful completion)

opt=(calcfc,ts,cartesian) mpw1pw91/6-31+g(d,p) geom=connectivity

Center Number	Atomic Number	Atomic Type	Coordinates (Angstroms) X	Y	Z
1	6	0	2.531896	-0.110979	2.576716
2	1	0	3.341088	0.211922	3.222888
3	6	0	2.453690	-1.455608	2.168628
4	1	0	3.203414	-2.164257	2.504313
5	6	0	1.431797	-1.881326	1.345798
6	1	0	1.389302	-2.919475	1.037841
7	6	0	0.421030	-0.982118	0.931980
8	6	0	0.508891	0.372459	1.342839
9	6	0	1.584919	0.795595	2.149575
10	1	0	1.645671	1.838805	2.443445
11	6	0	-0.474004	1.275690	0.820572
12	1	0	-0.381722	2.327120	1.081621
13	6	0	-1.793006	0.759670	0.588621
14	6	0	-1.893682	-0.595127	0.184781
15	6	0	-0.675626	-1.362344	0.078718
16	6	0	-3.170507	-1.109346	-0.134779
17	1	0	-3.278147	-2.145566	-0.432177
18	6	0	-4.293738	-0.312546	-0.039919
19	1	0	-5.269523	-0.730259	-0.263620
20	6	0	-4.182704	1.035634	0.345035
21	1	0	-5.070984	1.654522	0.411515
22	6	0	-2.944091	1.567800	0.640436
23	1	0	-2.846929	2.609011	0.932381
24	6	0	0.107874	1.348482	-1.204585
25	1	0	-0.692272	2.038109	-1.452150
26	6	0	0.067649	0.045020	-1.736981
27	1	0	-0.801761	-0.293463	-2.287703
28	6	0	1.417315	2.017936	-0.993421
29	6	0	2.625482	1.168429	-1.050761
30	1	0	3.554049	1.634831	-0.738005
31	6	0	2.568220	-0.092020	-1.505651
32	1	0	3.450135	-0.719344	-1.587453

33	6	0	1.302408	-0.691277	-1.995483
34	8	0	1.501016	3.218009	-0.767382
35	8	0	1.329319	-1.758462	-2.607707
36	6	0	-0.713475	-2.743352	-0.498693
37	1	0	0.247534	-3.015410	-0.938845
38	1	0	-1.457016	-2.827370	-1.291016
39	1	0	-0.957551	-3.479579	0.277109

9-MA + MA (TS)

opt=(calcfc,ts) freq mpw1pw91/6-31+g(d,p) geom=connectivity

Center Number	Atomic Number	Atomic Type	Coordinates (Angstroms) X	Y	Z
1	6	0	2.654819	1.453243	-1.883528
2	1	0	3.449866	2.150954	-2.123027
3	6	0	2.715451	0.133505	-2.369402
4	1	0	3.557567	-0.180043	-2.976462
5	6	0	1.715168	-0.762798	-2.063265
6	1	0	1.765426	-1.788767	-2.412330
7	6	0	0.606145	-0.351750	-1.295514
8	6	0	0.542005	0.976050	-0.797061
9	6	0	1.601254	1.863861	-1.095684
10	1	0	1.579160	2.878483	-0.717252
11	6	0	-0.428712	-1.251370	-0.888886
12	1	0	-0.353341	-2.286192	-1.213667
13	6	0	-1.735032	-0.713519	-0.649428
14	6	0	-1.808163	0.613732	-0.154679
15	6	0	-0.571111	1.329203	0.044638
16	6	0	-2.908933	-1.480879	-0.780971
17	1	0	-2.836145	-2.501207	-1.145112
18	6	0	-4.137545	-0.933409	-0.475144
19	1	0	-5.041548	-1.518766	-0.603820
20	6	0	-4.219417	0.388362	0.000123
21	1	0	-5.188121	0.818785	0.230202
22	6	0	-3.076801	1.142388	0.173861
23	1	0	-3.161408	2.159152	0.538806
24	6	0	0.028740	-0.179474	1.777636
25	1	0	-0.771195	0.236312	2.370702
26	6	0	0.073608	-1.447641	1.192647
27	1	0	-0.653142	-2.234371	1.338466
28	6	0	1.506843	-1.805658	1.070305
29	6	0	1.410817	0.266754	1.966309
30	8	0	2.256454	-0.706845	1.437896
31	8	0	2.015703	-2.831528	0.712474
32	8	0	1.852878	1.266340	2.466510
33	6	0	-0.573928	2.661123	0.734247
34	1	0	-0.772157	3.468780	0.019465
35	1	0	0.389769	2.853711	1.209445
36	1	0	-1.336269	2.709097	1.512319

9,10-DMA + BQ (TS)

opt=(calcfc,ts) freq mpw1pw91/6-31+g(d,p) geom=connectivity

Center Number	Atomic Number	Atomic Type	Coordinates (Angstroms) X	Y	Z
1	6	0	2.457637	0.702101	2.559493
2	1	0	3.216983	1.244672	3.112522
3	6	0	2.457654	-0.702552	2.559355
4	1	0	3.217016	-1.245212	3.112276
5	6	0	1.492494	-1.394899	1.858155
6	1	0	1.503764	-2.478221	1.864142
7	6	0	0.469965	-0.709674	1.164984
8	6	0	0.469951	0.709446	1.165117
9	6	0	1.492462	1.394561	1.858425
10	1	0	1.503703	2.477881	1.864627
11	6	0	-0.551191	1.391982	0.404657
12	6	0	-1.822362	0.708329	0.291116
13	6	0	-1.822349	-0.708445	0.290986
14	6	0	-0.551159	-1.392092	0.404388
15	6	0	-3.044850	-1.393325	0.132276
16	1	0	-3.060146	-2.476319	0.137257
17	6	0	-4.233036	-0.701619	0.003603
18	1	0	-5.167055	-1.244935	-0.091541
19	6	0	-4.233047	0.701520	0.003726
20	1	0	-5.167074	1.244838	-0.091324
21	6	0	-3.044873	1.393220	0.132525
22	1	0	-3.060180	2.476214	0.137694
23	6	0	0.128399	0.706154	-1.619880
24	1	0	-0.700964	1.235445	-2.075577
25	6	0	0.128483	-0.705947	-1.619955
26	1	0	-0.700789	-1.235284	-2.075766
27	6	0	1.406330	1.436450	-1.652450
28	6	0	2.634751	0.670575	-1.343155
29	1	0	3.535442	1.254643	-1.184052
30	6	0	2.634838	-0.670064	-1.343212
31	1	0	3.535603	-1.254031	-1.184157
32	6	0	1.406518	-1.436072	-1.652574
33	8	0	1.476484	2.630374	-1.929577
34	8	0	1.476830	-2.629964	-1.929799
35	6	0	-0.530736	-2.891553	0.326582
36	1	0	0.469036	-3.276746	0.129456
37	1	0	-1.168082	-3.258159	-0.478462
38	1	0	-0.887478	-3.324776	1.268816
39	6	0	-0.530801	2.891455	0.327098
40	1	0	0.468982	3.276717	0.130174
41	1	0	-0.887689	3.324514	1.269353
42	1	0	-1.168048	3.258169	-0.477975

9,10-DMA + MA (TS)

opt=(calcfc,ts) mpw1pw91/6-31+g(d,p) geom=connectivity

Center Number	Atomic Number	Atomic Type	Coordinates (Angstroms) X	Y	Z
1	6	0	-2.647207	0.702725	2.330858
2	1	0	-3.446845	1.244057	2.824564
3	6	0	-2.647340	-0.702534	2.330687
4	1	0	-3.447084	-1.243834	2.824257
5	6	0	-1.639288	-1.396347	1.697089
6	1	0	-1.657136	-2.479207	1.693777
7	6	0	-0.573374	-0.710547	1.073273
8	6	0	-0.573233	0.710608	1.073446
9	6	0	-1.639020	1.396484	1.697432
10	1	0	-1.656629	2.479350	1.694404
11	6	0	0.483180	-1.392565	0.368646
12	6	0	1.754579	-0.709912	0.305979
13	6	0	1.754719	0.709711	0.306193
14	6	0	0.483527	1.392613	0.369234
15	6	0	2.983232	-1.394230	0.187159
16	1	0	2.998715	-2.477359	0.195777
17	6	0	4.174016	-0.702463	0.099780
18	1	0	5.111222	-1.245117	0.038512
19	6	0	4.174159	0.701863	0.099986
20	1	0	5.111475	1.244345	0.038880
21	6	0	2.983517	1.393836	0.187576
22	1	0	2.999207	2.476961	0.196539
23	6	0	-0.098669	0.698349	-1.700989
24	1	0	0.661505	1.337193	-2.125627
25	6	0	-0.098970	-0.698763	-1.700525
26	1	0	0.660672	-1.338068	-2.125458
27	6	0	-1.507835	-1.128182	-1.705967
28	6	0	-1.507189	1.128499	-1.706578
29	8	0	-2.304446	0.000353	-1.594767
30	8	0	-1.987932	-2.227072	-1.783758
31	8	0	-1.986802	2.227568	-1.784903
32	6	0	0.434690	2.888836	0.243015
33	1	0	0.668047	3.366510	1.201775
34	1	0	-0.552844	3.223489	-0.077266
35	1	0	1.151495	3.251907	-0.494491
36	6	0	0.434235	-2.888833	0.242551
37	1	0	-0.553363	-3.223474	-0.077546
38	1	0	0.667733	-3.366439	1.201305
39	1	0	1.150895	-3.252000	-0.495054

Individually Plotted Data From Figure 3

Because some of the data in Figure 3 overlaps, we have included figures here in which each line is plotted individually.

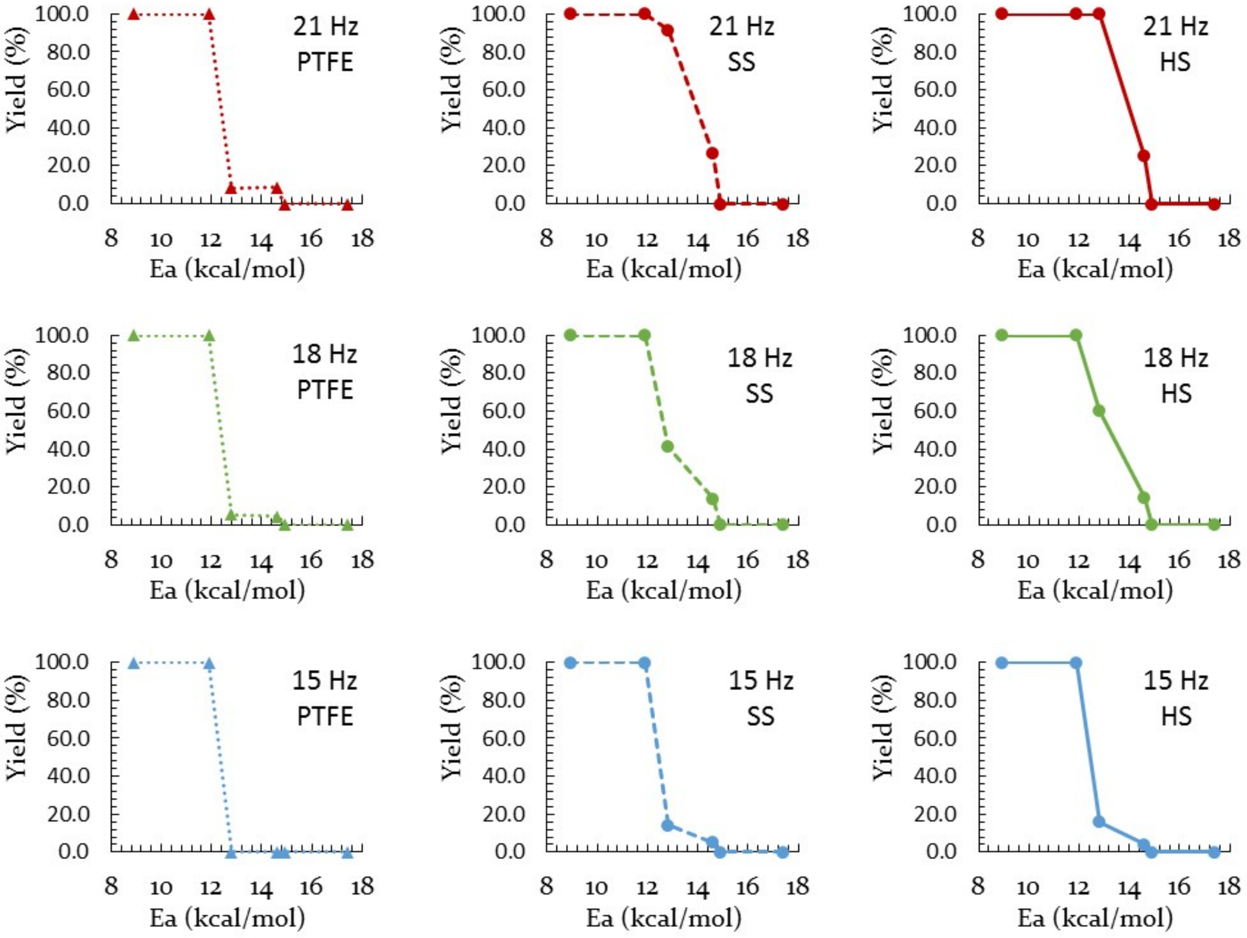

Supplement: Supplementary file 1 [file SC-008-C7SC00538E-s001.pdf]
